# *Leptospira* Seroprevalence in Domestic Dogs and Cats on the Caribbean Island of Saint Kitts

**DOI:** 10.1155/2017/5904757

**Published:** 2017-11-27

**Authors:** Nicola Pratt, Anne Conan, Sreekumari Rajeev

**Affiliations:** Department of Biomedical Sciences, Ross University School of Veterinary Medicine, Basseterre, Saint Kitts and Nevis

## Abstract

Leptospirosis is an important bacterial zoonotic disease that affects humans and many animal species. Knowledge of prevalence of* Leptospira* in a given geographic region supports the implementation of effective control/prevention programmes and thus reduces the transmission risks. This study provides* Leptospira* seroprevalence and serovar distribution in dogs and cats on the Caribbean island of Saint Kitts. Convenient serum samples from domestic dogs (*n* = 101) and cats (*n* = 50) were tested by the microscopic agglutination test (MAT) using 21* Leptospira* serovars belonging to 17 serogroups. Seroprevalence was recorded at 73.2% in dogs (95% confidence interval CI: 62.5–80.1%). Agglutinating antibodies to* Leptospira* were present against 13 of the 21 serovars tested by MAT. The highest seroprevalence was observed for serovar Autumnalis (56.4%) followed by Icterohaemorrhagiae (27.7%), Canicola (17.8%), Djasiman (14.9%), Bratislava (11.9%), Pyrogenes (11.9%), and Pomona (7.9%). A very low seroprevalence (4%, 95% CI: 0.5–14%) was observed in cats. This data confirms that dogs in Saint Kitts have a high-level exposure to a diverse set of* Leptospira* serovars.

## 1. Introduction

Leptospirosis is an important reemerging infectious disease with a broad host range and an important public health problem [1]. Leptospirosis is caused by the pathogenic members of the genus* Leptospira* that comprises more than 250 known serovars, conveniently grouped into serogroups based on the antigenic similarities [2]. Domestic and wild animals may act as reservoirs of pathogenic* Leptospira*, through asymptomatic colonization of the proximal renal tubules, and shed the organism in urine resulting in continual environmental contamination [3]. Infection is acquired after exposure to urine from infected animals or contact with environment or water contaminated with urine of the infected animals.

*Leptospira* infection in animals and humans may result in fatal outcomes from renal and hepatic disease and pulmonary haemorrhage. Canine leptospirosis presents with a spectrum of clinical illness ranging from mild illness to fatal life-threatening disease similar to humans. Icteric and/or anicteric illness with renal, hepatic, and pulmonary manifestations occurs in dogs [4]. The members of the serogroup Icterohaemorrhagiae, Canicola, Grippotyphosa, Pomona, and Australis have been reported in canine infections [4, 5]. Seroprevalence is observed to be less in cats; however, the evidence of renal carriage is reported and higher seropositivity rates in cats with chronic kidney disease have been described [6, 7].

Leptospirosis is endemic in the Caribbean region and high-level human mortality and morbidity are estimated [8, 9]; however, animal studies are limited in the region. To the author's knowledge, the only* Leptospira* seroprevalence data from animals published from Saint Kitts dates back to 1996, conducted in livestock species [10]. There are two reports of canine leptospirosis from Saint Kitts [11, 12]. To date, no* Leptospira* seroprevalence data is available from domestic dogs and cats from the island. This study was aimed at obtaining preliminary* Leptospira* seroprevalence data from dogs and cats on the island of Saint Kitts.

## 2. Materials and Methods

### 2.1. Area of Study

The island of Saint Kitts is located in the Caribbean region and is one of the Leeward Islands in the Lesser Antilles. Saint Kitts has a tropical climate with an average rainfall estimated at around 125 cm (50 inches) to 200 cm (80 inches) with the wettest season from May to October. The average temperature varies from 23°C to 31°C (http://www.stkittstourism.kn/).

### 2.2. Dog and Cat Serum Samples

This study utilized convenient serum samples banked in the Ross University School of Veterinary Medicine (RUSVM), Veterinary Diagnostic Laboratory, collected from dogs and cats presented to the Ross University Veterinary Clinic (RUVC) during a period of 2014-2015. Random samples were selected and tested blindly.

### 2.3. Microscopic Agglutination Test

The presence of agglutinating anti-*Leptospira* antibodies in canine serum samples was tested by microscopic agglutination test (MAT) using a panel of 21* Leptospira* serovars (listed in Table 1) originally obtained from the US Centres of Disease Control and Prevention (CDC) in Atlanta, Georgia, USA, and maintained in the laboratory through weekly subcultures [13]. The* Leptospira* serovars included in the MAT panel were subcultured regularly and incubated at 29°C in EMJH (Ellinghausen-McCullough-Johnson-Harris) media.* Leptospira* subcultures for the MAT were used between the 4th and 7th day of incubation, at a transmittance measurement of 60–70% at 400 nm, in accordance with standard operating protocols of the RUSVM* Leptospira* laboratory. Serum samples were initially screened at a final dilution of 1 : 100 using a dark-field microscope equipped with a long 5x objective and a dry dark-field condenser. Samples showing 50% agglutination at a dilution of 1 : 100 were recorded as positive. All positive samples were then serially diluted and tested to determine the antibody titer. Homologous positive serum samples for each of the serovars tested (obtained from Royal Tropical Institute, Netherlands) and negative controls were included in the assay as a test quality control measure.

### 2.4. Data Analysis

A descriptive statistical analysis was performed. Seroprevalence and exact 95% confidence interval (95% CI) were computed. For canine samples, origin of the dog (imported or local) and leptospirosis vaccination status were retrieved. Comparisons of seroprevalence between groups as well as association between serovars were done using a nonparametric Fisher test. Threshold of significance was set at a *p* value of 0.05. Data analyses were performed using R software [14] and the package epiDisplay [15].

## 3. Results

We tested 50 serum samples from cats and 101 serum samples from dogs collected during the years 2014-2015. In dogs, overall seroprevalence was of 73.2% (95% CI: 62.5–80.1%) (Table 2). Exposure to 14 serovars out of 21 tested was observed. Of all the canine serum samples (*n* = 101), 31 serum samples (31%) were found to react to only one serovar, and 42 samples (42%) were found to react to more than one serovar. Out of these samples, 17 reacted to two serovars, 12 reacted to three serovars, 4 reacted to four serovars, 4 reacted to five serovars, 1 reacted to six serovars, and 3 reacted to seven serovars. The highest rate of seroprevalence was observed to serovar Autumnalis followed by serovar Icterohaemorrhagiae. The seropositivity to serovar Autumnalis is significantly associated with positive results to serovar Icterohaemorrhagiae (*p* = 0.0003), Djasiman (*p* = 0.001), Pomona (*p* = 0.009), and Pyrogenes (*p* = 0.01). The majority of positive reactions (*n* = 150, 87%) had recorded titer values of 1 : 100 or 1 : 200. Observed MAT titers for various serovars are given in Table 3. The MAT titers ranged from 1 : 100 to 1 : 6400 and the highest titer recorded was 1 : 6400 to serovar Icterohaemorrhagiae in one of the dogs.

Of the 101 canine samples tested, 58 animals (57%) were local island dogs, 26 (26%) were imported to the island mainly from USA, and 17 dogs had no information available on origin and/or vaccination status. Vaccinated category is the dogs that have received a* Leptospira* vaccine at any time or they are current on* Leptospira* vaccination based on the available patient records. There was no significant difference in seroprevalence between groups based on origin (*p* = 1) (Table 4). The seroprevalence in all vaccinated dogs in the study was found to be 56.5% (13/23) and in all nonvaccinated dogs 69.2% (54/78). Seroprevalence in all local dogs was 70.7% (41/58), with seroprevalence in nonvaccinated local dogs found to be 72.3% (34/47). In vaccinated local dogs, the seroprevalence was recorded at 61.5% (8/13). Of all imported dogs, the seroprevalence was 73.1% (19/26), with nonvaccinated and vaccinated imported dogs having seroprevalence of 78.8% (15/19) and 50.0% (5/10), respectively. The difference in seroprevalence in vaccinated dogs (56.6%) and nonvaccinated dogs (69.2%) was not found to be statistically significant (*p* = 0.3) (Table 4). No significance difference in seroprevalence was observed between vaccinated and nonvaccinated dogs to serovars included in the vaccine (Table 5). In this study, the difference in seroprevalence between male and female dogs was not statistically significant (*p* = 0.4).

A very low seroprevalence was observed in cats (4%, 95% CI: 2.2–19.2%; *N* = 2/50). The only detected serovars were Cynopteri (2%, 95% CI: 0.5–13.7%; *N* = 1/50) and Pomona (2%, 95% CI: 0.5–13.7%; *N* = 1/50). A low titer (1/100) was recorded for both serovars.

## 4. Discussion

The study performed on the island of Saint Kitts provides information on the general seroepidemiology of* Leptospira* infection in domestic dogs and cats. The low seroprevalence rate in cats is in agreement with other serosurveys conducted on cats which have generally shown rates of seroprevalence to vary from around 5% to 12%, with outdoor cats tending to have higher rates of seroprevalence [16, 17]. An older study in Caribbean region has recorded a prevalence of 12.5% (5/40) in cats on the island of Trinidad [18].

A high seroprevalence to multiple serovars was observed in dogs and suggests a high degree of* Leptospira* exposure. The overall seroprevalence in dogs recorded on Saint Kitts (72.3%) is higher than that recorded in Puerto Rico (62.9%) [19] predominantly to serogroups, Icterohaemorrhagiae, Andamana, and Pyrogenes, in Barbados with a seroprevalence of 62%, predominantly to serogroups Australis, Icterohaemorrhagiae, and Autumnalis [20], and in Trinidad (15.5%) predominantly to serogroups Icterohaemorrhagiae and Copenhageni the predominant serovar [21]. Six canine serum samples had titer values greater than or equal to 1 : 800. In areas where* Leptospira* are endemic, a titer value greater than 1 : 800 is indicative of active infection in humans when appropriate clinical signs are present [22]. In our study, 12 canine serum samples gave a positive reaction to serovar Bratislava, but none was positive to serovar Australis and both belong to the same serogroup. It has been suggested that dogs may be an alternative maintenance host for the serovar Bratislava [23]. A seropositive reaction to the serogroup Australis has been associated with severe clinical disease in dogs [24–26].

One of the limitations of this study is the sample size and the choice of canine serum samples selected at random, from the diagnostic laboratory at RUSVM without knowledge of the reason for presenting at the clinic and lack of access to other clinical data. The high titers observed in some serum samples could have been due to the selection of serum from potential undiagnosed leptospirosis cases. In other studies, conducted on other islands, when comparisons were made between healthy animals and clinically ill animals,* Leptospira* seroprevalence was greater in the dogs suspected of having leptospirosis than in the healthy group of dogs [20, 21, 27]. This was not an option in this study as only data on sex, vaccination status, and origin was available. It has been reported previously that male dogs have a greater risk of infection [28]. However, our study and another study [29] did not find any significant association between sex or age and* Leptospira* seroprevalence.

The prevalence of agglutinating antibodies to* Leptospira* serovars varies widely around the globe and even within countries. Worldwide, high* Leptospira* seroprevalence in dogs has been shown to serovars Icterohaemorrhagiae, Canicola, Grippotyphosa, and Bratislava but these may vary with the region [3]. Seroprevalence patterns have been changing especially in areas where vaccines containing serovars Canicola and Icterohaemorrhagiae are used [30–32]. Antibody prevalence to serovar Autumnalis is the highest in our study and a similar observation has been reported in other studies mentioned above. In the absence of* Leptospira* isolations belonging to this serovar from dogs in the above-mentioned study regions, cross-reactivity to other serovars such as Pomona is speculated [30]. In our study, positive results to Autumnalis were associated with positive results to serovars Icterohaemorrhagiae, Pomona, Djasiman, and Pyrogenes. Therefore, it is difficult to conclude whether this is due to exposure to members of serogroup Autumnalis, or cross-reactivity. However, serovar Bim belonging to the serogroup Autumnalis has been isolated from toads and frogs from Barbados [33–35]. In addition, an isolate belonging to this serogroup isolated from a dog is listed in the National Veterinary Services* Leptospira* catalogue (unpublished data). Therefore, it is possible that* Leptospira* strains belonging to the serogroup Autumnalis may be present in the water and environment and dogs are exposed to this serovar in the Caribbean region. This needs further confirmation by actively pursuing isolation of circulating serovars, though a laborious activity is ongoing in our laboratory. It is important to consider that cross-reactivity between multiple serovars may also result from paradoxical reactions described in recent infections [13].

MAT is described to provide prevalence information at the serogroup level in epidemiological investigations. In our study, it is important to note the lack of consistency in reactivity between serovars of the same serogroup. Twenty-eight samples reacted to serovar Icterohaemorrhagiae where only 14 reacted to serovar Mankarso and both serovars belong to the same serogroup. A similar inconsistency was found within serogroups Pyrogenes and Australis used in this study. In our quality control procedures, cross-reactivity is clear in reaction to homologous positive control rabbit sera and the members of the same serogroups. The reactivity in MAT is based on the outer membrane antigens, and the observed lack of cross-reactivity might be due to low antibody titers or variation in antigen expression during natural infections. Our data suggests that including only a representative member of the serogroup in the MAT panel may underestimate the serological prevalence. The use of strains at serogroup level in the MAT panel needs further refinement.

This study has shown the presence of agglutinating antibodies to 13 of the 21 serovars in canine serum samples used in the MAT panel, indicating a potential exposure to a large and diverse pool of* Leptospira* serovars. A high diversity of serogroups has been shown to be present in equatorial and/or tropical areas and is believed to be related to the presence of a wide range of mammalian reservoir hosts and a suitable environment that favors the survival of* Leptospira* [36].

Vaccination is the main strategy for prevention of leptospirosis in dogs. The clinic at RUSVM uses the Vanguard L4 vaccine which contains serovars Canicola, Icterohaemorrhagiae, Pomona, and Grippotyphosa. Lack of significant correlation associated with vaccinated and nonvaccinated population needs further investigations. This study indicates that the number of serovars that dogs can potentially be exposed on the island is large and vaccination using commercial vaccines may not always be successful, as the current vaccines do not provide cross protection to other serovars that are not included in the vaccine. The positive results on MAT testing may be due to natural infection and exposure or through vaccination. The data concerning the date of vaccination and the serovars present in the vaccine used was unavailable for imported dogs. In addition, the prevalent serovars in dogs observed in our study do not totally reflect the shift in serovars seen in other areas of the world. Consequently, the vaccine protocol used on the island may not be comparable to that used in other places and may not provide effective protection from disease. No statistical difference in prevalence was observed between island dogs and imported dogs. Island dogs were found to be positive to a greater number of serovars. The level of exposure may be high in the free roaming island dogs compared to imported dogs owned by the expatriate population that mostly remain indoors.

Infected dogs, like other mammals, can be potential source of zoonosis to their owners and may result in severe disease and/or death in those in contact. Dogs may be used as a sentinel species for prediction of changes in seroprevalence and environmental contamination [26]. We recently observed that* Leptospira* seroprevalence is high (49.4%) among African Green Monkeys inhabiting the island [37]. A difference in exposure was observed between wild (38.3%) and captive monkeys (60.5%) with predominant exposure to serovars Bratislava (27.2%) and Ballum (16.0%) in wild monkeys and serovars Bataviae (45.7%) and Ballum (21.0%) in captive monkeys. It is important to note that the exposure to serovars Bataviae and Ballum was absent in the canine population tested indicating that the source of infection and risk factors for these two highly exposed groups are different.

It is intriguing that the seroprevalence is low in cats compared to dogs, where both these species are inhabiting an island with high* Leptospira* endemicity and cats are the main scavengers for the rodent species, a well-known reservoir of pathogenic* Leptospira*. While difference in lifestyle of domestic cats and dogs may provide a simple explanation, the role of other complex factors including difference in immune system and genetics needs further exploration.

## 5. Conclusions

While serology has some limitations, it is still the most widely used method to assess* Leptospira* prevalence. This preliminary study and the first one of this kind on the island of Saint Kitts has shown a high* Leptospira* seroprevalence among dogs on the island. The exposure to a range of serovars detected in dogs is an indication of the possible existence of a variety of animal and environmental sources. The high* Leptospira* seroprevalence in dogs observed in this study suggests a high infection pressure to susceptible hosts from the environment and consequently a high risk of infection for animals and humans.* Leptospira *isolation from the environment and animal reservoirs must be undertaken in addition to the serology tests in order to better understand the epidemiology and transmission of infection in the Caribbean region.

## Figures and Tables

**Table 1:** Serovar panel used in the microscopic agglutination test.

Species	Serogroup	Serovar
*L. interrogans*	Icterohaemorrhagiae	Icterohaemorrhagiae
*L. interrogans*	Icterohaemorrhagiae	Mankarso
*L. interrogans*	Autumnalis	Autumnalis
*L. interrogans*	Pyrogenes	Pyrogenes
*L. santarosai*	Pyrogenes	Alexi
*L. interrogans*	Bataviae	Bataviae
*L. interrogans*	Grippotyphosa	Grippotyphosa
*L. interrogans*	Canicola	Canicola
*L. weilii*	Celledoni	Celledoni
*L. kirschneri*	Cynopteri	Cynopteri
*L. interrogans*	Djasiman	Djasiman
*L. santarosai*	Mini	Georgia
*L. borgpetersenii*	Tarassovi	Tarassovi
*L. interrogans*	Sejroe	Hardjo
*L. interrogans*	Sejroe	Wolffi
*L. borgpetersenii*	Javanica	Javanica
*L. interrogans*	Australis	Australis
*L. interrogans*	Australis	Bratislava
*L. interrogans*	Pomona	Pomona
*L. borgpetersenii*	Ballum	Ballum
*L. interrogans*	Hebdomadis	Borincana

**Table 2:** Seroprevalence to *Leptospira* serovars in dogs in Saint Kitts collected during the years 2014-2015. Samples were randomly selected from storage bank and blinded from the tester.

Serovars	Number of samples	Number of positive samples	Seroprevalence (%)	95% confidence interval (%)
Autumnalis	101	57	56.4	46.2–66.3
Icterohaemorrhagiae	101	28	27.7	19.3–37.6
Canicola	101	18	17.8	10.9–26.7
Djasiman	101	15	14.9	8.6–23.3
Mankarso	101	14	13.9	7.8–22.26
Bratislava	101	12	11.9	6.3–19.8
Pyrogenes	101	12	11.9	6.3–19.8
Pomona	101	8	7.9	3.5–15.0
Hardjo	101	3	3.0	0.6–8.4
Alexi	101	2	2.0	0.2–7.0
Celledoni	101	1	1.0	0.03–5.4
Cynopteri	101	1	1.0	0.03–5.4
Grippotyphosa	101	1	1.0	0.03–5.4

**Table 3:** The number of dogs with MAT titers to various *Leptospira* serovars.

Serogroup	Serovar	1 : 100	1 : 200	1 : 400	1 : 800	1 : 1600	1 : 6400
Icterohaemorrhagiae	Icterohaemorrhagiae	25	2				1
	Mankarso	2	6	3	3		
Grippotyphosa	Grippotyphosa	1					
Canicola	Canicola	14	1	3	1		
Celledoni	Celledoni	1					
Cynopteri	Cynopteri	1					
Pomona	Pomona		5	3			
Djasiman	Djasiman	7	7		1		
Pyrogenes	Pyrogenes	9	2	1			
	Alexi	2					
Autumnalis	Autumnalis	42	9	5		1	
Australis	Bratislava	3	8	1			
Sejroe	Hardjo	2	1				

**Table 4:** Seroprevalence in dogs, based on vaccination status and origin.

Groups	Number of samples	Number of positive samples	Seroprevalence (%)	95% confidence interval (%)		*p* value (Fisher test)
Origin						
Local	58	41	70.7	57.3	81.9	1
Imported	26	19	73.1	52.2	88.4	
Unknown origin	17	3	—	—	—	—
Vaccination status						
Vaccinated	23	13	56.5	34.4	76.9	0.3
Nonvaccinated	78	54	69.2	57.8	79.2	
Vaccination status in local dogs						
Vaccinated	13	8	61.5	31.6	86.1	0.5
Nonvaccinated	45	33	73.3	58.1	85.4	
Vaccination status in imported dogs						
Vaccinated	6	4	66.7	22.2	95.7	1
Nonvaccinated	20	15	75.0	50.9	91.3	

**Table 5:** Seroprevalence in vaccinated and unvaccinated dogs to serovars present in the vaccine used in the clinic.

Serovars	Prevalence in vaccinated group (%) [95% confidence interval]	Prevalence in nonvaccinated group (%) [95% confidence interval]	*p* value (Fisher's test)
Icterohaemorrhagiae	21.7 [3.5–40.0]	31.5 [20.6–42.4]	0.4
Pomona	4.3 [0–13.4]	9.6 [2.7–16.5]	0.7
Canicola	13.0 [0–27.9]	20.5 [11.1–30.0]	0.5
Grippotyphosa	0	0.01 [0–0.04]	1
